# Centralized industrialization of pork in Europe and America contributes to the global spread of *Salmonella enterica*

**DOI:** 10.1038/s43016-024-00968-1

**Published:** 2024-05-09

**Authors:** Heng Li, Yilei Wu, Dan Feng, Quangui Jiang, Shengkai Li, Jie Rong, Ling Zhong, Ulrich Methner, Laura Baxter, Sascha Ott, Daniel Falush, Zhenpeng Li, Xiangyu Deng, Xin Lu, Yi Ren, Biao Kan, Zhemin Zhou

**Affiliations:** 1 Key Laboratory of Alkene-Carbon Fibres-Based Technology & Application for Detection of Major Infectious Diseases, MOE Key Laboratory of Geriatric Diseases and Immunology, Pasteurien College, Suzhou Medical College, Soochow University, Suzhou, China; 2 Suzhou Key Laboratory of Pathogen Bioscience and Anti-infective Medicine, Jiangsu Province Engineering Research Center of Precision Diagnostics and Therapeutics Development, Soochow University, Suzhou, China; 3 Department of Biological Sciences, Xi’an Jiaotong–Liverpool University, Suzhou, China; 4 Institute of Bacterial Infections and Zoonoses, Friedrich-Loeffler-Institut, Jena, Germany; 5 Warwick Bioinformatics Research Technology Platform, University of Warwick, Coventry, UK; 6 Warwick Medical School, University of Warwick, Coventry, UK; 7 The Center for Microbes, Development and Health, CAS Key Laboratory of Molecular Virology and Immunology, Shanghai Institute of Immunity and Infection, Chinese Academy of Sciences, Shanghai, China; 8 National Key Laboratory of Intelligent Tracking and Forecasting for Infectious Diseases, National Institute for Communicable Disease Control and Prevention, Chinese Center for Disease Control and Prevention, Beijing, China; 9 Center for Food Safety, University of Georgia, Griffin, GA USA; 10 Iotabiome Biotechnology Inc., Suzhou, China

**Keywords:** Pathogens, Data mining, Evolutionary genetics

## Abstract

*Salmonella enterica* causes severe food-borne infections through contamination of the food supply chain. Its evolution has been associated with human activities, especially animal husbandry. Advances in intensive farming and global transportation have substantially reshaped the pig industry, but their impact on the evolution of associated zoonotic pathogens such as *S. enterica* remains unresolved. Here we investigated the population fluctuation, accumulation of antimicrobial resistance genes and international serovar Choleraesuis transmission of nine pig-enriched *S. enterica* populations comprising more than 9,000 genomes. Most changes were found to be attributable to the developments of the modern pig industry. All pig-enriched salmonellae experienced host transfers in pigs and/or population expansions over the past century, with pigs and pork having become the main sources of *S. enterica* transmissions to other hosts. Overall, our analysis revealed strong associations between the transmission of pig-enriched salmonellae and the global pork trade.

## Abstract (web summary)

By analysing the genome of over 9,000 pig-associated isolates, this study shows that modernized agricultural systems have favoured the acquisition of antimicrobial resistance genes, population expansion and global transmission of pig-enriched *Salmonella* over the past century.

## Main

*Salmonella enterica* infiltrates food supply chains through the contamination of food, water or food-processing facilities^1^, resulting in life-threatening food-borne infections with 108.1 million illnesses and 291,000 deaths annually^2^. Pork and pigs are prominent sources of *S. enterica* infections, accounting for ~31.1% of salmonellosis and 9.3% of disease outbreaks in the European Union^3^. Despite the acknowledged role of pigs in mediating transmissions and outbreaks of viral diseases^4,5^, their contribution to the global dissemination of bacterial pathogens, including *S. enterica*, remains insufficiently explored within the framework of the ‘One Health’ strategy.

The developments of intensive farming and global trade over the past century have drastically transformed pig agriculture^6^, giving rise to two industrial hubs, Europe and the United States, that collectively represent >32% of international pork and pig breed trades^7,8^. While most of the >2,000 populations in *S. enterica*, recognized by their serovars, eBGs (eBurst Groups) or ceBGs (cgMLST eBGs) based on the genomic sequences^9^, are ubiquitous, some populations predominantly comprise strains associated with pigs^10^ and are found to spread regionally through movements of pigs and/or wild boars^11^. Nonetheless, it remains unclear how pathogens, especially these pig-enriched salmonellae, have disseminated globally and how their population dynamics have been recrafted by modern agriculture.

## Results

### Landscape of pig-enriched *S. enterica* populations

**Fig. 1:**
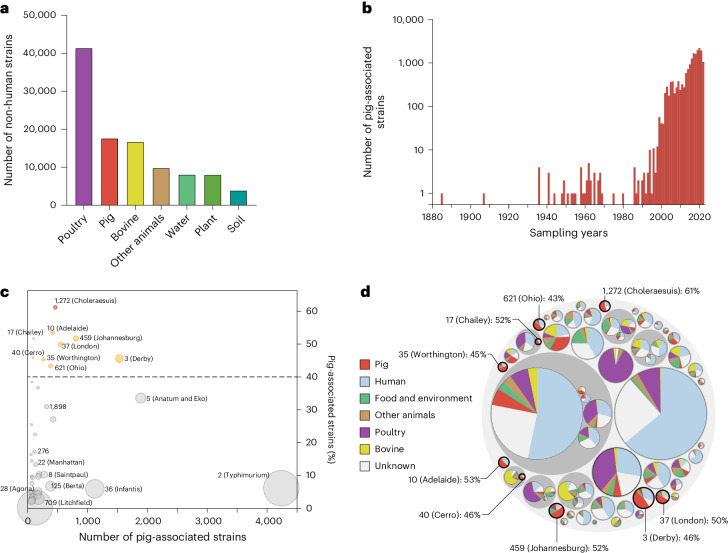
Summary of the pig-enriched ceBGs in the *Salmonella* database in EnteroBase. **a**, Histogram of the numbers of *Salmonella* strains for each non-human source in EnteroBase. **b**, Histogram of the numbers of pig-associated *Salmonella* strains per year in 1885–2022. **c**, Bubble plot of the 61 pig-associated ceBGs, each with ≥20 strains. Each ceBG is proportional in size to the number of strains in it and placed according to the numbers and percentages of its pig-associated strains. **d**, Hierarchical bubble plot of the 61 pig-associated ceBGs as in **c**. The sizes of the circles are proportional to the number of strains, and the three levels in the plot represent (from outer to inner) clusters at the levels of subspecies (HC2850), super-lineage (HC2000) and ceBG (HC900), as described previously^9^. Pie charts represent the proportions of strains from different sources. The 9 pig-enriched ceBGs, each with >40% pig strains, are labelled in **c** and **d**. Source data

Systematic investigation of all 362,931 *Salmonella* strains publicly accessible in EnteroBase (July 2022) showed that pigs and pork account for 17,623 strains (4.9%) in 252 ceBGs and are the second most frequent livestock source of *S. enterica* after poultry over the past century (Fig. 1a,b). There are 61 major ceBGs, each containing ≥20 pig-related strains, of which 9 have ≥40% of their strains from pigs and pork (Fig. 1c,d and Supplementary Table 5), including prominent pig-enriched populations such as ceBG1272 (Choleraesuis) and ceBG3 (Derby), and others such as ceBG10 (Adelaide) and ceBG459 (Johannesburg). Pigs are the primary sources (4,393; 49%) of strains in these 9 pig-enriched ceBGs, followed by humans (2,482; 28%) and other animals (1,279; 14%). The other ceBGs have lower levels of pig-associated strains (0.2–38%) and exhibit no clear host preference, including ceBG2 (Typhimurium), which has only 6% (4,612) of pig-associated strains (Fig. 1c,d).

The HC5 clusters in EnteroBase, namely, clusters of strains with ≤5 allelic differences in their core genes, have been extensively used in epidemiological investigations for designating genetically almost identical bacteria such as those from disease outbreaks^12^. Unexpectedly, while most HC5s in the pig-enriched ceBGs are from single countries, there are 35 HC5s each consisting of strains from ≥2 countries, including 15 HC5s with strains from different continents (Supplementary Table 1), indicating very recent international or even cross-continental transmissions. Notably, all these international HC5s contain at least one pig strain, indicating the importance of pigs for these long-range transmissions.

### Europe as the main genetic repository of serovar Choleraesuis

**Fig. 2:**
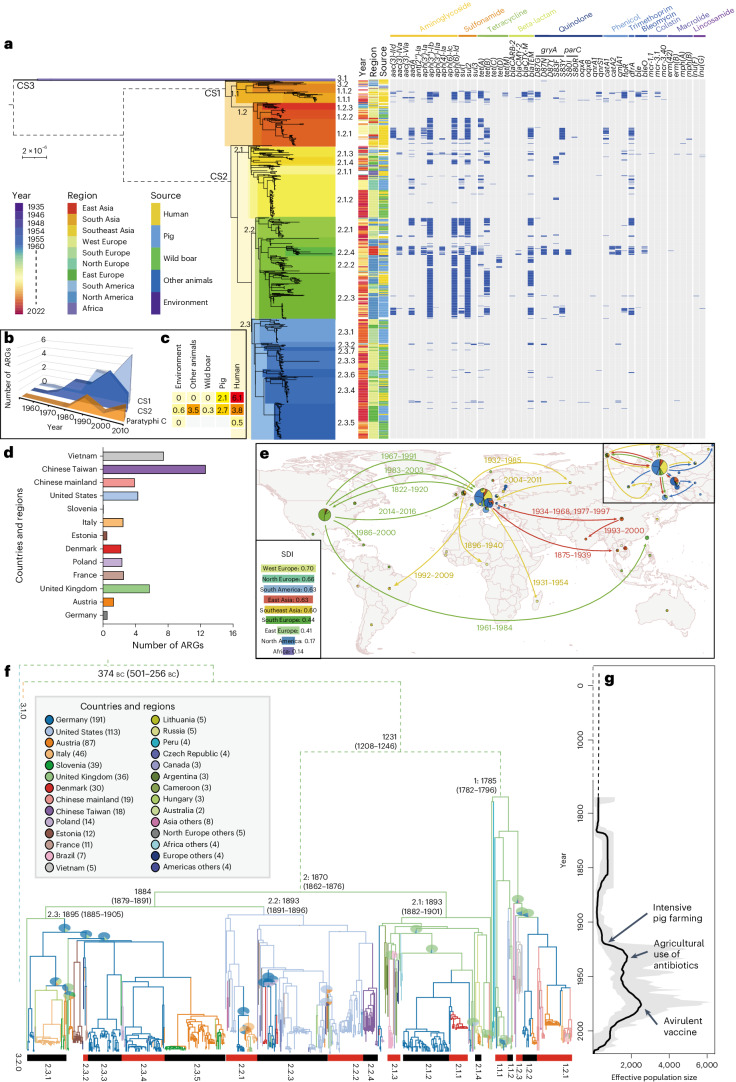
Population dynamics, ARGs and global transmissions of ceBG1272 (Choleraesuis). **a**, The maximum-likelihood phylogeny (left), metadata (middle) and predicted ARGs for all strains in ceBG1272 (right). The predicted lineages, clades and clusters are labelled near the associated branches. The associations between the numbers of predicted ARGs and the sampling years, source categories and countries are also visualized. **b**,**c**, The associations between the numbers of predicted ARGs and the sampling years in Paratyphi C lineages (**b**) and the association between the number of ARGs per strain and host sources (**c**). **d**, Visualization of the correlations between the numbers of predicted ARGs and countries in Choleraesuis. **e**–**g**, Bayesian inferences of the population dynamics of ceBG1272 over the past ~2,500 years. **e**, Global transmission of ceBG1272. Pie charts show the proportional composition of clades in each major country, and the arrows show the transmissions reconstructed based on the tree in **f**, with the transmission dates shown nearby. The pie charts and arrows were colour coded based on the associated clades. Inset: the Simpson diversity of clades in each geographic region in the world. **f**, The MCC tree of ceBG1272 by BEAST 2. The branches were colour coded based on the most probable ancestral geographic origins (as in the key). Pie charts of all possible geographic origins are shown over certain nodes where the most probable origins had <90% posterior supports. The dates of origin for some branches are shown together with the 95% confidence intervals in brackets. **g**, The fluctuation of effective population sizes with time by the ‘skygrowth’ package in R. Arrows point to the time of three major developments in the modern pig industry. Credit: Map in **c**, Santiago H. Cardona (https://github.com/hrcarsan/world-map/blob/master/LICENSE). Source data

**Extended Data Fig. 1:**
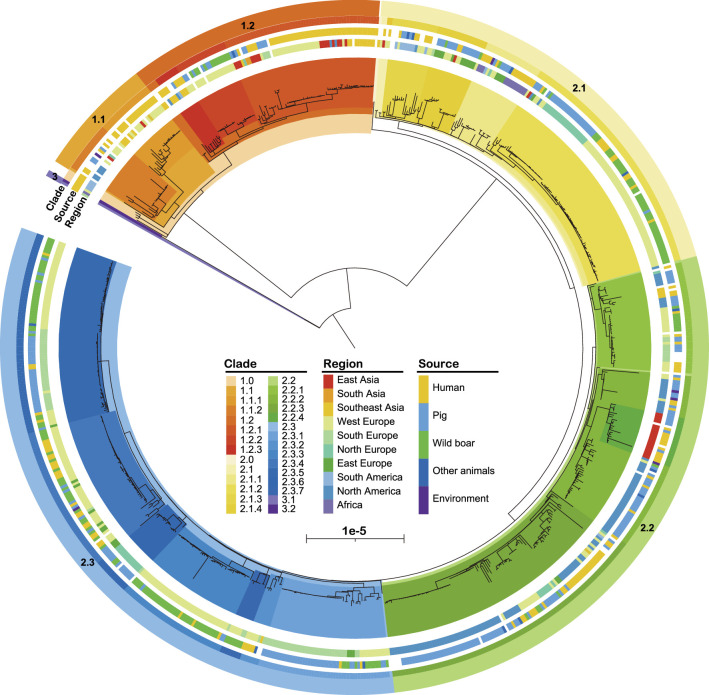
Circular presentation of the maximum-likelihood phylogeny in Fig. 1a. Outer rings: The clade, source, and geographic origin of each strain. Colored arcs underneath the tree show the cluster assignments as in the key. Source data

We reconstructed a maximum-likelihood phylogeny of serovar Choleraesuis based on 21,948 non-repetitive, non-recombinant single nucleotide polymorphisms in the core genome and used it to divide strains into three lineages of CS1 CS2, and CS3 from the root that were separated by 2,932 to 12,435 single nucleotide polymorphisms (Supplementary Table 6). Except for CS3, which contained only two strains, the other two lineages were subdivided into clades and clusters (Fig. 2a and Extended Data Fig. 1). CS1 consists of 8 clusters in two clades, CS1.1 and CS1.2, and CS2 consists of 19 clusters in three clades, CS2.1 to CS2.3. High geographic and host specificities were found in certain clades and clusters. For example, most of the strains in the Chinese mainland and Vietnam fell into clade 1.2, while many of the British strains were from clade 1.1. In addition, 92% of the US strains grouped with those from Chinese Taiwan in clade 2.2, while >60% of European wild boar strains were from clade 2.3.

We evaluated the genetic diversity of Choleraesuis in different regions worldwide. Simpson’s diversity index (SDI) for the presence of clades in different regions (Fig. 2e) showed that the West and North European regions had the greatest levels of diversity (0.66–0.7), followed by South America and East Asia (0.63). By contrast, Africa and North America had the lowest SDIs (0.14 and 0.17, respectively). Furthermore, to minimize the impact of oversampling in developed countries, we performed a country-level comparison, which also showed that the countries from North Europe exhibited greater levels of SDIs than those from other regions (Supplementary Table 2). This suggested that Europe was the main genetic repository and probably the origin of serovar Choleraesuis (Fig. 2e).

### Host-specific antimicrobial resistance gene accumulation in Choleraesuis

Choleraesuis strains carried many more antimicrobial resistance genes (ARGs) than its human-specific analogue, Paratyphi C (Fig. 2b,c), which was separated from serovar Choleraesuis only ~4,000 years ago, suggesting an association between hosts and ARGs. Furthermore, significant differences in ARG levels were found between Choleraesuis strains from different hosts, countries and regions. In particular, strains from humans and livestock carried approximately ninefold more ARGs than those from wild boars (Fig. 2c), and strains from Chinese Taiwan and Vietnam carried more ARGs than those from others (Fig. 2d).

Moreover, Choleraesuis isolated from pigs exhibited high resistance against aminoglycoside, sulfonamide, tetracycline and beta-lactam, which were all common feed supplements for intensive farming^12^ (Fig. 2a). By contrast, resistances against clinical antimicrobials including quinolone, trimethoprim and cephalosporins were much fewer and often found only in human strains. Notably, the colistin resistance genes *mcr-1* and *mcr-3* commonly detected in pigs^13^ were found in eight human and pig strains from the UK, China, Brazil and Germany, underscoring their global presence (Supplementary Table 3). In addition, continuous increases in ARG carriages over time were spotted in CS1 and CS2 strains isolated after the 1970s (Fig. 2b), but the ARG carriages in CS2 dropped after the 2010s owing to an increase of strains from wild boars.

### International transmission of serovar Choleraesuis

**Extended Data Fig. 2:**
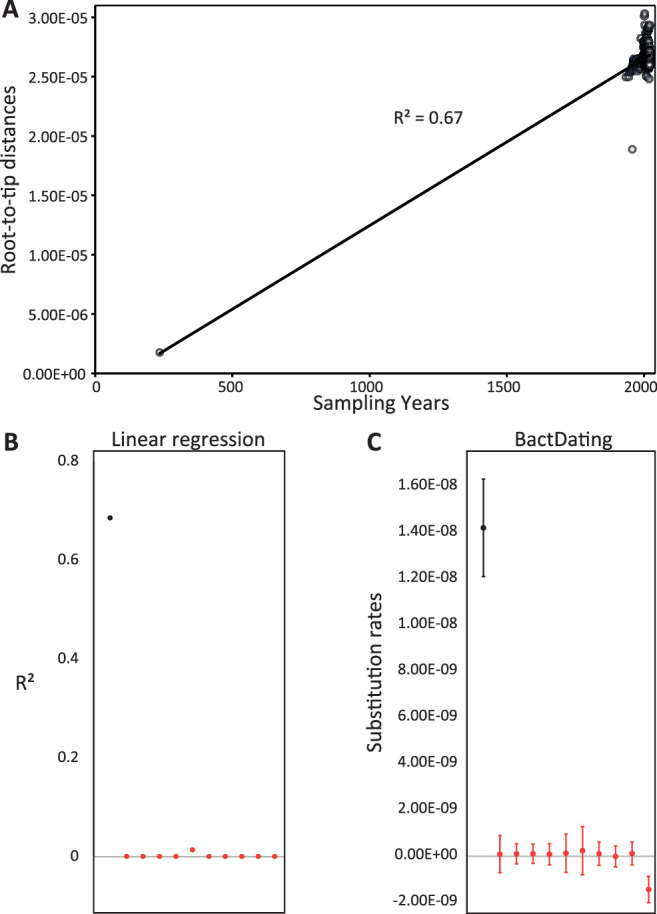
Evaluation of the presence of temporal signal in *S. enterica* serovar Choleraesuis. The analysis was performed on 587 genomes. **a**, Linear regression between root-to-tip distances of strains and the sampling years with a coefficient of determination (R^2^) of 0.67. **b**, Substantially lower R^2^ values were obtained for ten date-randomisation datasets. **c**, The average (dots) and standard deviation (error bars) of the substitution rates for actual data (black) and ten date-randomization datasets (red), estimated by BactDating. Source data

**Extended Data Fig. 3:**
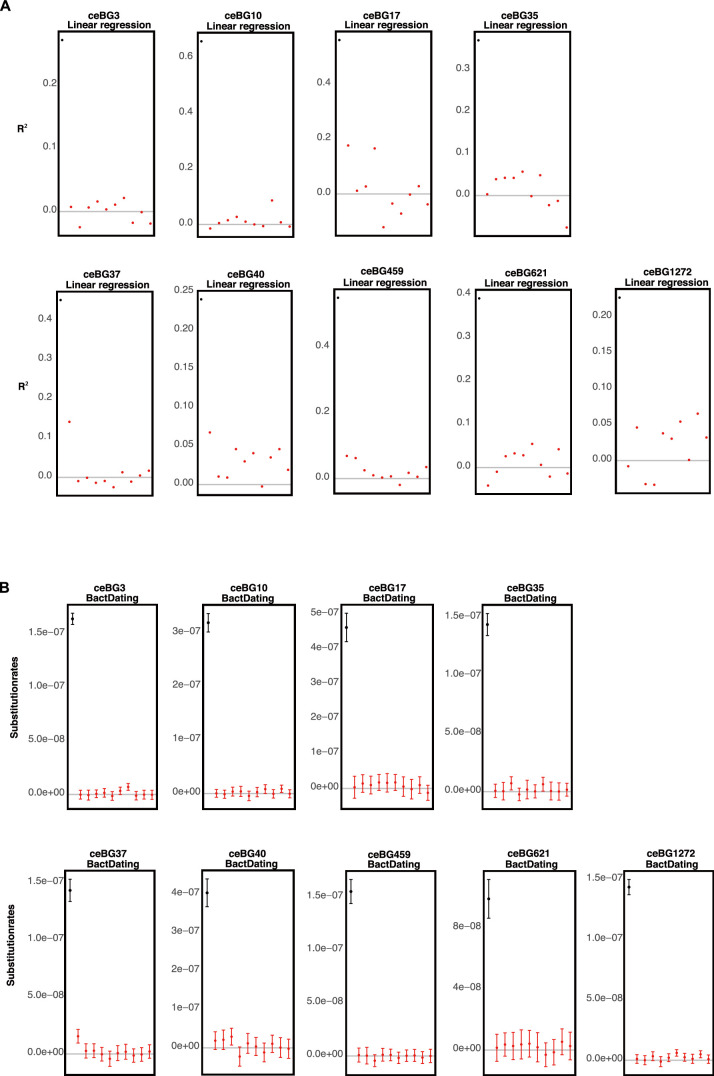
Assessing the existence of temporal signal randomization test in pig-enriched ceBGs. **a**, Coefficients of determination (R^2^) for the ten date-randomisation tests were obtained by linear regression between root-to-tip distances of strains and the sampling years in pig-enriched ceBGs. **b**, The average (dots) and standard deviations (error bars) of the substitution rates for actual data (black) and ten date-randomization datasets (red) by BactDating. Pig-enriched ceBGs including ceBG3 (n = 3136), ceBG10 (n = 622), ceBG17 (n = 155), ceBG35 (n = 516), ceBG37 (n = 1012), ceBG40 (n = 176), ceBG459 (n = 1441), ceBG621 (n = 787), ceBG1272 (n = 586). The ancient sample was not included in ceBG1272 in both **a** and **b**. Source data

Significant temporal signals were detected in serovar Choleraesuis, with and without the ancient genotype (Extended Data Figs. 2 and 3). Bayesian inferences predicted that the most recent common ancestor (MRCA) of Choleraesuis had probably been circulating in Europe before 2394 bp (95% CI 2276–2521 bp) and diverged there into CS1 and CS2 in 1785 and 1870, respectively (Fig. 2f). The first predicted transmission outside of Europe occurred before 1893 (95% CI 1891–1896) and resulted in clade CS2.2 in the United States. Soon after, the effective population size of Choleraesuis was predicted to experience an expansion in the early twentieth century, coincident with the rapid development of intensive pig farming, and reached its first peak in the 1930s, before the commercial use of synthetic sulfonamides and other antimicrobials in animals^14^.

After 20 years of stale, a second expansion of Choleraesuis was predicted between the 1950s and the 1980s (Fig. 2g). The frequencies of international transmissions also increased, possibly associated with the rapid expansion of global agricultural trade as part of the post-war waves of livestock revolution and trade globalization^15^. Europe and the United States were the major sources of international transmissions. For example, European CS1 strains were repetitively transmitted to the Chinese mainland and Southeast Asia (Fig. 2f). Furthermore, CS2.2 strains were transmitted from the United States to Chinese Taiwan (Fig. 2f, CS2.2.4) in 1962 (95% CI 1957–1968) and became endemic there for more than 50 years, causing major human outbreaks between 1996 and 2002^16^. The population size of Choleraesuis reached its peak in 1985 and underwent continuous decreases afterwards (Fig. 2g). This was also accompanied by a decrease in long-range transmissions, although the local transmissions in Europe remained frequent, partially attributed to the movement of wild boars^17^.

**Extended Data Fig. 4:**
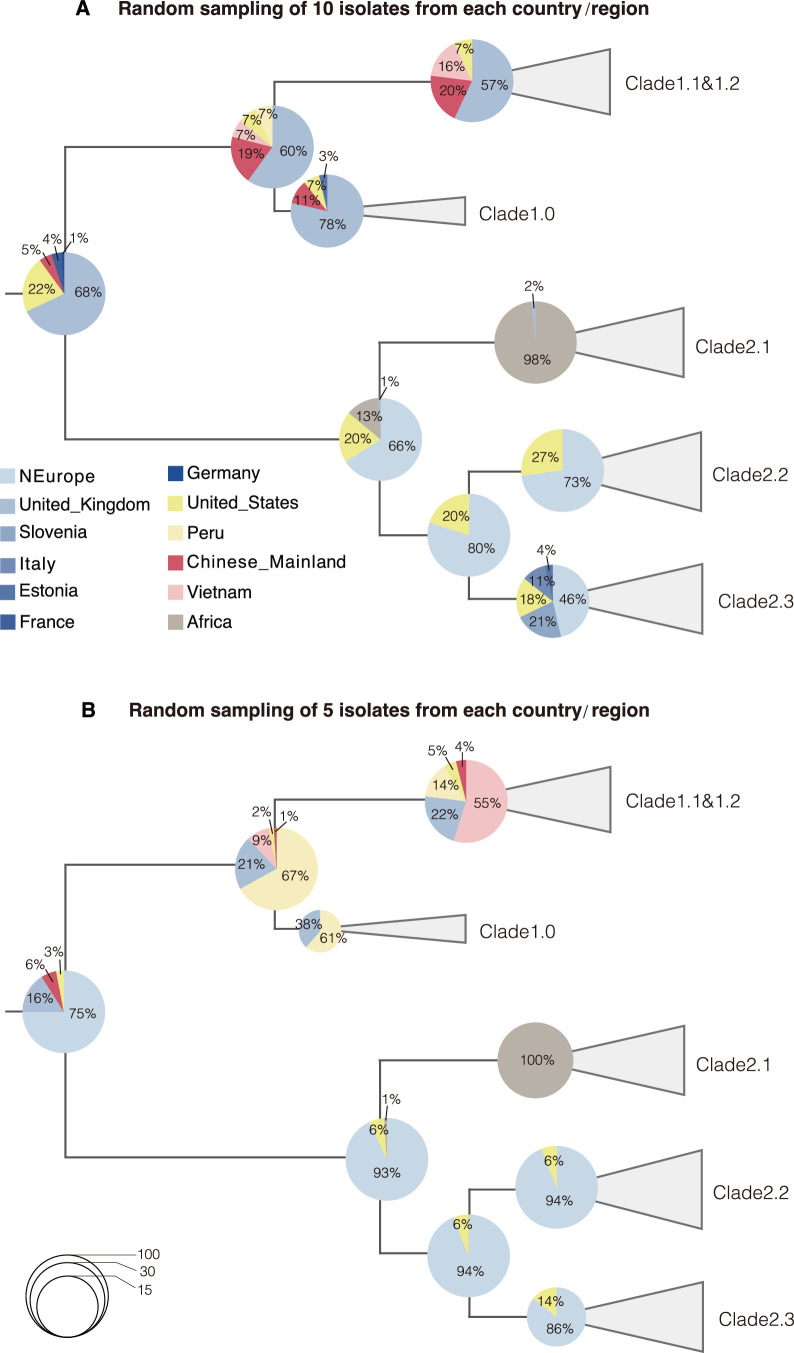
The geographic states of the ancestral nodes in the Choleraesuis population after downsamplings. **a**, Downsampling tests of up to ten strains per country/region. **b**, Downsampling tests of up to five strains per country/region. Both: the geographic states were predicted using TreeTime. Each test was run in 100 parallels. The pie charts illustrate the proportions of the best-supported geographic states in the 100 parallels for the corresponding ancestral nodes. Source data

The majority of the Choleraesuis genomes were isolated from North European countries, which could lead to sampling bias in the analyses. Therefore, we performed phylogeographic reconstructions by downsampling at most ten random genomes from each country (Extended Data Fig. 4 and Supplementary Table 4). Summarizing 100 downsampling results together, we found that the MRCA of Choleraesuis and the MRCAs of all major clades except for clade 2.1 were still from North Europe (Extended Data Fig. 4a). Downsampling to at most five genomes per country still proposed North Europe as the origin of the whole population but made Peru the origin of lineage 1 (Extended Data Fig. 4b). These differences probably resulted from the fact that there was only one cluster of strains for each of Peru and Cameroon near the basal of clade 2.1 and lineage 1, respectively.

### Intensive farming in establishments of pig-enriched serovars

**Fig. 3:**
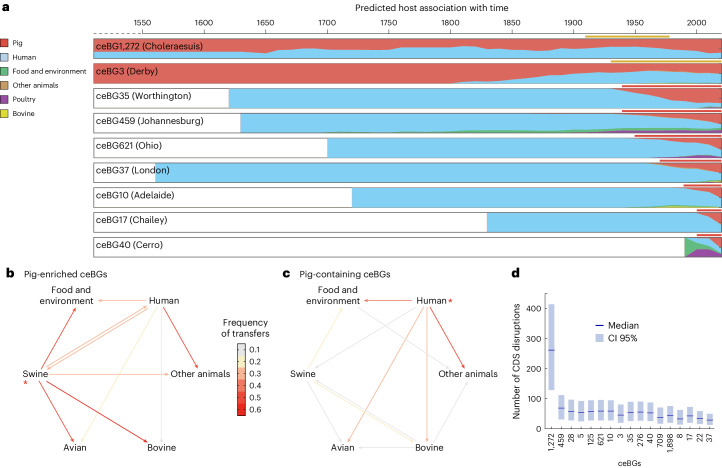
Host transfers for the pig-enriched ceBGs. **a**, The curves show the dynamic changes of the proportional host sources with time for each ceBG. The ancestral host associations were predicted by TreeTime. The predicted median effective population sizes are also shown as black curves for ceBG1272 (Choleraesuis) and ceBG3 (Derby). The period for host transfers into pigs (red) or population expansions (yellow) is shown above each plot. A detailed prediction of the population dynamics for all nine ceBGs can also be found in Extended Data Fig. 5. **b**, Proportional source of host transfers summarized for all nine ceBGs in the past 50 years. Detailed host transfer data for each ceBG can be found in Extended Data Fig. 6. **c**, Proportional source of host transfers in the past 50 years summarized for eight pig-containing ceBGs that have 5–35% of pig strains, including ceBGs of 5, 8, 22, 125, 276, 709 and 1,898 (Fig. 1c). **b**,**c**, The arrows show the direction of transfers and are colour coded by average frequencies. The sources with the most contributions are indicated with asterisks. **d**, Median numbers of disrupted coding sequences (CDSs) per genome with 95% confidence intervals in all pig-enriched and pig-containing ceBGs. Source data

**Extended Data Fig. 5:**
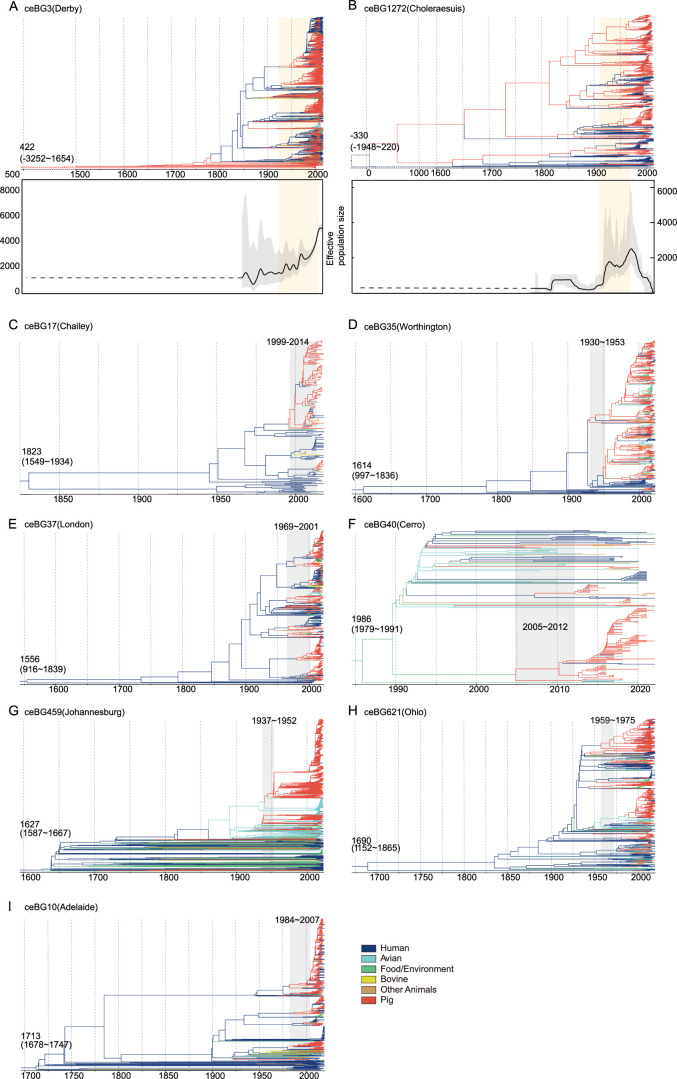
Reconstructed ancestral host associations for all nodes in nine pig-enriched ceBGs. The corresponding ceBGs for each panel are: **a**, ceBG3 (Derby), **b**, ceBG1272 (Choleraesuis), **c**, ceBG17 (Chailey), **d**, ceBG35 (Worthington), **e**, ceBG37 (London), **f**, ceBG40 (Cerro), **g**, ceBG459 (Johannesburg), **h**, ceBG621 (Ohio), **i**, ceBG10 (Adelaide). The mean values of effective population sizes with time were also shown for **a** and **b**, with 95% confidence intervals in grey shapes. The orange and grey boxes show the periods of population expansion (**a**,**b**) or the periods of host transfers into pigs (**c**-**i**). Source data

We further evaluated the role of pigs in the evolution of all nine pig-enriched populations. To this end, we showed the presence of temporal signals in all nine populations by date randomizations (Extended Data Fig. 3) and estimated their temporal phylogenies and ancestral host transfers. Notably, apart from ceBG40 (Cerro), which originated around 1954, the MRCAs for other populations were all predicted to be present before the nineteenth century (Fig. 3a and Extended Data Fig. 5). However, except for ceBG3 (Derby) and ceBG1272 (Choleraesuis), other populations were originally present in hosts other than pigs, and transferred into pigs only after 1930. Furthermore, we evidenced at least eight human-to-pig transmissions in ceBG3 (Derby) in 1906–1942, resulting in the establishment of the contemporary pig-enriched lineages and major population expansion (Extended Data Fig. 5a). Thus, all pig-associated salmonellae, apart from Choleraesuis, probably experienced host transfers in the twentieth century (Fig. 3a). Furthermore, the accumulation of pseudogenes has been associated with a drastic change, such as host adaptation of the bacteria. However, we did not find evidence of an accumulation of pseudogenes in any population (Fig. 3d), except for the pig-adapted Choleraesuis, which has >17.3% of its genes disrupted^7^.

**Extended Data Fig. 6:**
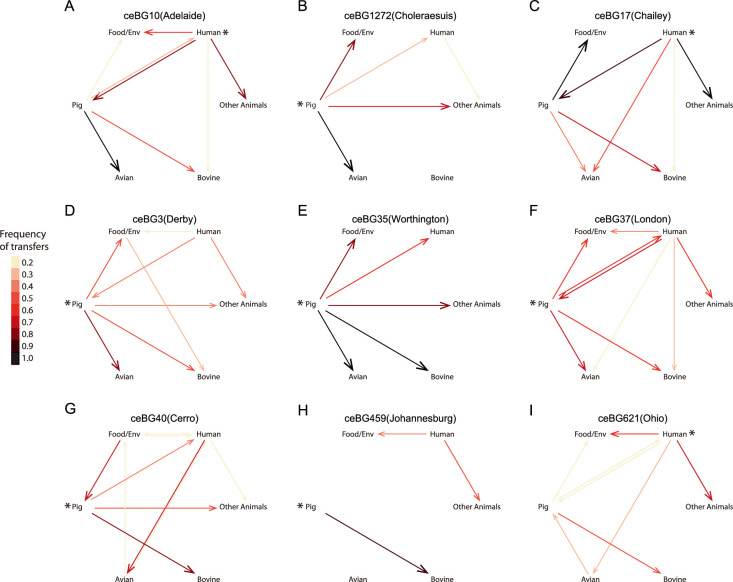
The host transmission frequency of nine pig-enriched ceBGs. The corresponding ceBGs for each panel are: **a**, ceBG10 (Adelaide), **b**, ceBG1272 (Choleraesuis), **c**, ceBG17 (Chailey), **d**, ceBG3 (Derby), **e**, ceBG35 (Worthington), **f**, ceBG37 (London), **g**, ceBG 40 (Cerro), **h**, ceBG 459 (Johannesburg), **i**, ceBG 621 (Ohio). Different arrows represent the direction of host transmission, with darker colors indicating higher transfer frequency. “*” marks the most contributing host sources for the transmissions. Source data

Different from the long-term trend of human-to-pig transfers, the majority (30–60%) of the recent host transfers in these populations, including those into humans, have been contributed by pigs in the past 50 years (Fig. 3b and Extended Data Fig. 6). Conversely, pigs contribute much less to host transfers in eight populations that had lower proportions (7–38%) of pig strains (Fig. 3c), showing the importance of pigs from intensive farming as a hub of host transfers in the pig-enriched populations.

### Dispersal of *S. enterica* from Europe and America

**Fig. 4:**
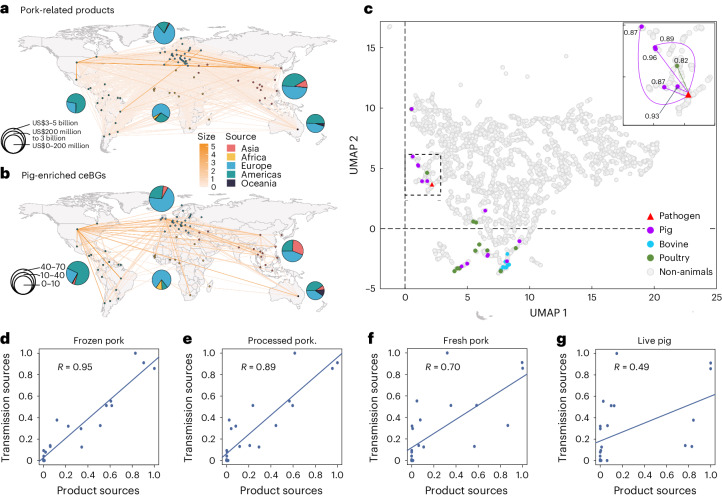
Association between the transmission of the pig-enriched ceBGs and the global trade of pig-related products. **a**,**b**, Visualization of the international trade of all pork-related products (**a**) and the transmissions of pig-enriched ceBGs (**b**). The pie charts show the relative proportions of source continents for the products or pathogens to the target continents. **c**, UMAP plot of Pearson’s correlations among the trade and the transmission of pig-enriched salmonellae. Each coloured dot in the plot shows an animal-related product as in the Harvard database, and the grey dots are other, non-animal, products. The triangle shows intercontinental transmission data summarized from all pig-enriched salmonellae. The insert highlights the dashed box in the plot, with the arrows specifying the correlation coefficient (*R*) between the transmissions of pathogens and the trade of pig-related products. **d**–**g**, Linear regressions of different categories of pig-related products (*x*-axis) and the intercontinental transmissions of pig-enriched ceBGs (*y*-axis). The correlation coefficient values for the linear regressions are also shown. Credit: Maps in **a**,**b**, Santiago H. Cardona (https://github.com/hrcarsan/world-map/blob/master/LICENSE). Source data

**Extended Data Fig. 7:**
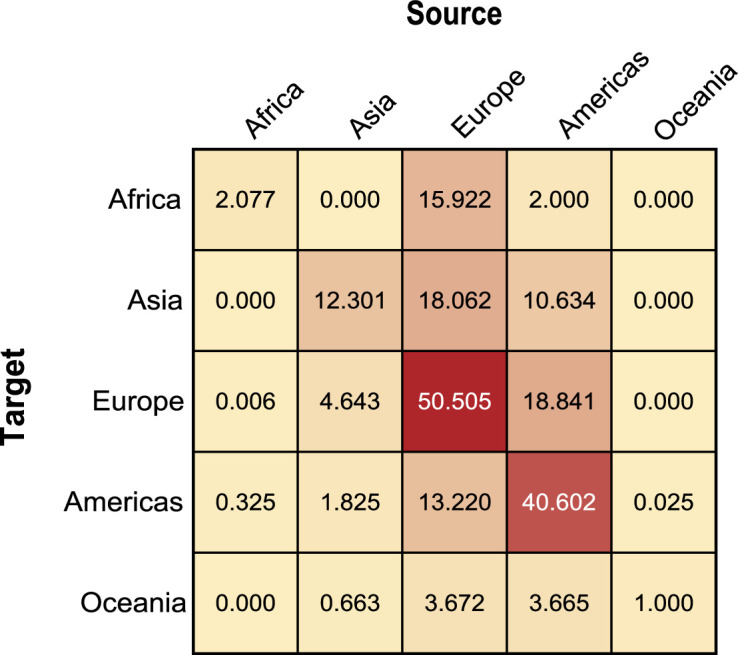
A 5 ×5 table showing the summarised international transmission events of all nine pig-enriched ceBGs in *Salmonella* in the past 50 years. The maximum-likelihood phylogeny was reconstructed based on SNPs in the core genome of each ceBG and the date of origin was estimated using BactDating. TreeTime was applied to reconstruct the country sources for all nodes in the tree. A transmission was recorded when the ancestral node and descending node of a branch were different. All transmissions were then summarized and grouped based on their associated continents. Source data

The reconstructed international transmissions in all nine populations showed that 68–96% of transmissions into each continent were from either Europe or America (Fig. 4a), exhibiting similar patterns to the trade data of the pork-related products in the Harvard database (Fig. 4b). We then summarized the cross-continental trades for each of the 5,014 product categories in the Harvard database. The transmission of pig-enriched salmonellae was shown in a 5 × 5 table, in which rows and columns represented the continental sources and targets, and each cell showed the percentage contribution of a source continent to the influx of the target (Extended Data Fig. 7). This resulted in a dataset for trade and transmissions, of which the pairwise Pearson’s correlations were calculated and projected to a two-dimensional space using an unsupervised method, the uniform manifold approximation and projection (UMAP^18^; Fig. 4c).

**Fig. 5:**
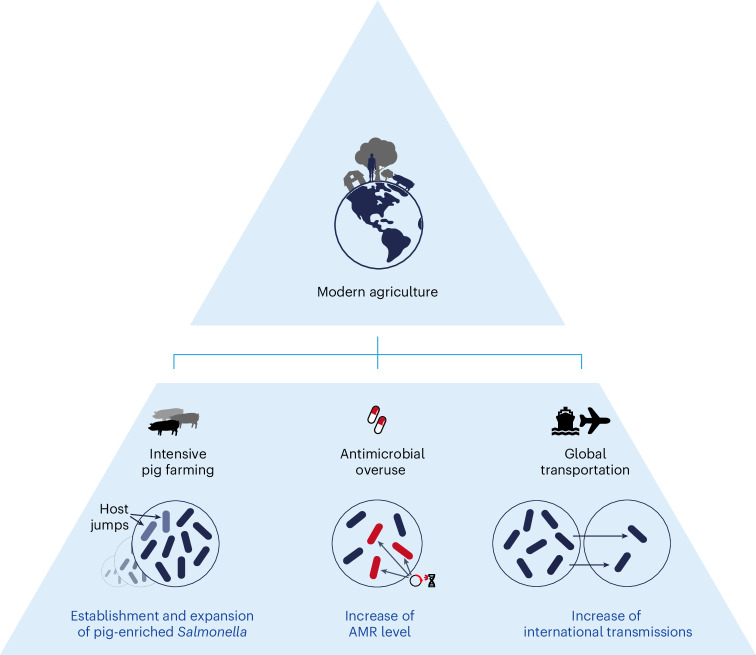
The influences of modern agriculture on the population dynamics of *S. enterica* serovars. This image provides a visual summary of the paper. Agricultural production has become increasingly modernized over the past half century. On the one hand, the pattern of large-scale intensive pig farming has led to the emergence and population expansion of pig-enriched *Salmonella*; on the other hand, globalized trade exchanges concerning pigs have similarly increased the probability of global transmission of pig-enriched *S. enterica* serovars. In addition, with the use of antibiotics in the process, more and more pig-enriched *Salmonella* has obtained new antibiotic resistance genes, including many of the previously reported human-specific antibiotic resistance genes. The impact of the development model of modern agriculture on pig-enriched *Salmonella* is comprehensive and far reaching. Credit: pill, pig and globe icons adapted from The Noun Project under a Creative Commons license CC0 1.0.

**Extended Data Fig. 8:**
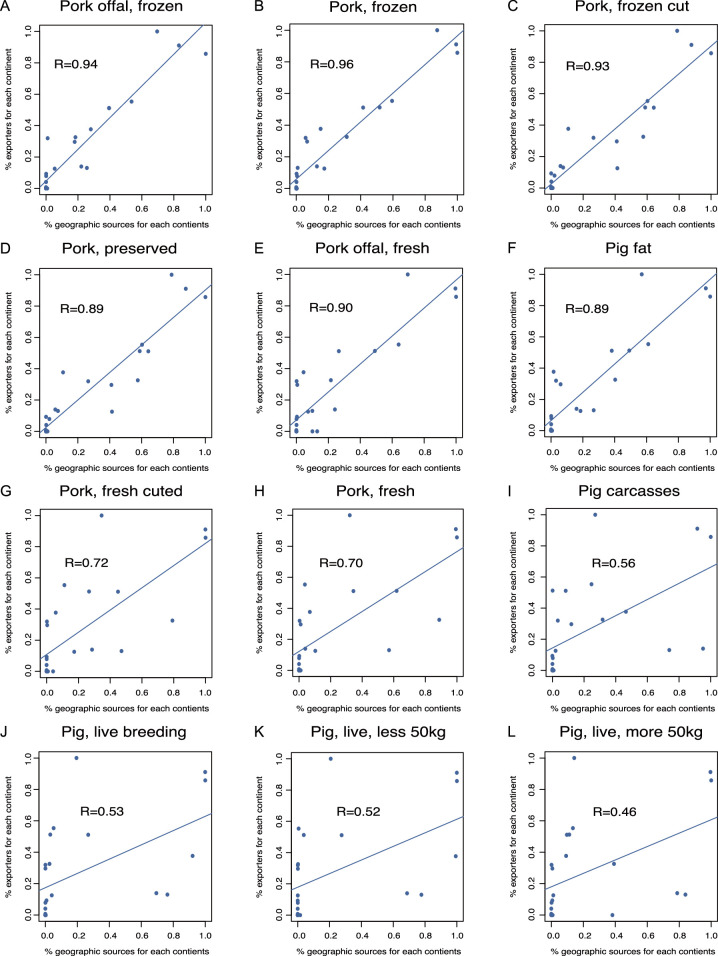
Pearson’s correlations analysis of sub-classification for pig-related products. The Sub classifications involved include **a**, Pork offal, frozen (021011); **b**, Pork, frozen (020649); **c**, Pork, frozen cut (020329); **d**, Pork, preserved (020322); **e**, Pork offal, fresh (020630); **f**, Pig fat (020910); **g**, Pork, fresh cuted (020912); **h**, Pork, fresh (020319); **i**, Pig carcasses (020310); **j**, Pig, live breeding (010391); **k**, Pig, live, less 50 kg (010392); **l**, Pig, live, over 50 kg (020311). The codes in parentheses represent the Harmonized System Codes of the products. Analyzing the correlation between the intercontinental dissemination of each pig-related commodity and the intercontinental transmission of pig-enriched ceBGs. R: Pearson’s correlation coefficient. Source data

The trade data for most of the animal-related products fell into two clusters in the projection. The first cluster consisted of almost all bovine and poultry products as well as live pigs, and the second cluster consisted of five pig-related products and one poultry product. Impressively, the transmission of the pig-enriched salmonellae also fell in the second cluster, exhibiting 0.87–0.96 correlation coefficients to the pig-related products (*P* < 0.0001; Fig. 4c). A detailed investigation of the products in the second cluster suggested that they were either frozen or processed pork or offal and fat that could be transported over long distances and used for pig feeding^19^. Pig-enriched salmonellae exhibited greater correlation with these products than with fresh pork and live pigs (Fig. 4d–g), which also correlated with pathogen transmissions with lower, yet significant, coefficients of 0.46–0.72 (Fig. 5 and Extended Data Fig. 8).

## Discussion

The modern agriculture system, including intensive farming and global transportation, has significantly recrafted the daily life of not only livestock animals but also ourselves^6^. But how much has it modified the life of bacteria, particularly the zoonotic pathogens? Here, based on genomic analysis of >9,000 pig-associated strains, we showed that the modernization and globalization of agriculture in the past century had driven the emergence, population expansions, ARG acquisitions and global transmissions of pig-enriched salmonellae.

We initiated the investigation in Choleraesuis, a prominent serovar that has been specifically infecting both pigs and humans for >2,000 years^11^. Compared with previous studies that focused on either its ancient divergence or regional dissemination^11^, this study has compiled a global dataset of Choleraesuis strains to give a comprehensive overview of its recent evolution. We showed the high occurrences of cross-continental transmissions, with a peak in the 1950s to the 1980s, a period of accelerated globalization before the use of specific vaccines^20^. The most obvious example is between the Chinese mainland and Taiwan. Despite their geographic closeness, almost all strains in the Chinese mainland were in clade 1.2 and imported from Europe, whereas strains in Chinese Taiwan were in cluster 2.2.4 and imported from the United States. We attributed this to the different trading partners between the two regions. The Chinese mainland mostly imported pork and pig breeds such as Yorkshires and Landraces from Europe in 1950–2000^21^, while Chinese Taiwan traded more frequently with the United States^22^.

The quinolone-resistant *Salmonella* has been regarded as an ‘urgent threat’ and is widely found in Typhi and Paratyphi A^23^. We found that 38% and 10.8% of Choleraesuis strains from humans and pigs were also quinolone resistant, mediated by either mutation in quinolone-resistance-determining regions (QRDR) or the acquisition of ARGs (Supplementary Table 6). Furthermore, many quinolone-resistant strains, especially the human strains in clusters 1.1.1, 1.2.1 and 2.2.4, also exhibit resistance to many antimicrobials extensively used in animals and clinical settings, and pose imminent threats to public health. For example, the strains from cluster 2.2.4 have been epidemic in Chinese Taiwan in 1995–2003^16^. Particularly, compared with those from wild boar, strains from pigs and humans had 10× more ARGs or QRDR mutations, revealing a strong association between the extensive animal use of antimicrobials and the emergence of extensively resistant pathogens^24^ and showing the importance of the ‘One Health’ strategy in the control of antimicrobial overuse.

Nine pig-enriched *Salmonella* populations were identified from the ~362,000 genomes in EnteroBase. Some Typhimurium clades were previously reported as pig adapted based on small, local datasets^25^, but not in our survey of >70,000 genomes (Fig. 1c). By contrast, we showed that two prominent pig-associated serovars, ceBG1272 (Choleraesuis) and ceBG3 (Derby), have been associated with pigs for millennia^26,27^ and experienced significant population expansions after the 1900s. In addition, the other seven pig-enriched populations were not associated with pigs until the 1900s, during which they all experienced host jumps into pigs. We noticed the development of intensive pig farming during the beginning of the 1900s, which could increase pig–pig contacts while reducing pig–human contacts. Such an environment could increase the chance of transmission among the pig populations while reducing spillover between the hosts, facilitating the establishment of host-enriched pathogens. Similar host transfer and population expansion have been observed in *Mycobacterium tuberculosis*, which was associated with increased contact between humans after the invention of fire use^28^. Thus, we attributed the increased host jumps and population expansion of salmonellae in the pig population to the development of intensive pig farming in the twentieth century, but further study is needed.

We showed that the vast majority of the contemporary non-human strains from these nine populations were from pigs and pork, and proved that pigs are the primary source of their host transfer events (Fig. 3b). The chance of strains from these nine pig-enriched populations being transmitted by other non-human hosts is low. Thus, we hypothesized that pig-related routes, including pork and pigs, the major sources of *S. enterica* infections^29^, were the dominant routes of transmission of these salmonellae. International salmonellosis outbreaks due to global transportation of end products have been extensively reported, such as those caused by contaminated hams^30^ or chocolates^31^. However, most of these events resulted in infections in humans, which represents a sink of the pathogen and rarely mediated population expansion or secondary transmissions into animals^32^. By contrast, transmissions via poultry breeding stocks^33^ or animal feeds^30^ could lead to long-term epidemics or permanent establishment of the bacteria in the target regions. Furthermore, the transmission of pig pathogens has been more frequently associated with the end products owing to the common application of swill feeding^19^, as evidenced in foot-and-mouth disease virus outbreaks^4^, African swine fever virus (ASFV)^5^ and trichinosis^34^. Based on the UMAP analysis, we revealed strong associations between the global pork trade and the transmissions of pig-enriched salmonellae. This indicated that the predominance of modern pig industries in Europe and the Americas made them the centres of development and global dissemination of salmonellae, highlighting the role of agricultural practice as a driver of the geographic dispersal of associated bacterial pathogens.

A limitation of this work is that the majority of involved genomes were from public databases and had sampling bias towards developed countries. The genetic diversities of pig-enriched salmonellae in the majority of developing countries, especially those in South America and Africa, have not been sampled. Downsampling was adopted to reduce the influence of such bias, but, as evidenced in Choleraesuis, could introduce a bias towards countries of low genetic diversities. Furthermore, there is not enough data for the investigation of local transmissions driven by country-wide agricultural transportation or the movements of wild boars, as previously reported^35^.

In summary, our findings show the influence of modern agriculture on the population dynamics of *S. enterica*. The intensive farming has driven the host jumps of many *S. enterica* populations into pigs and population expansion of the pig-associated populations, the widespread availability of antibiotics after the 1940s increased the prevalence of antimicrobial resistance (AMR), and the expansion of globalized trade and transportation resulted in rapid and frequent global dissemination of these pig-enriched *S. enterica* (Fig. 5). Despite decades of significant progress on *Salmonella* control in pigs, the evidence provided here warrants further investigation and potential intervention into the global spread of *S. enterica* from centralized origins at the pinnacle of pork production.

## Methods

### Strains and whole-genome sequencing procedures

The metadata associated with all 362,931 *S. enterica* strains accessible in EnteroBase (July 2022) were downloaded and manually classified into seven categories: pig, bovine, poultry, human, other animals, and food and environment. The serovar associated with each ceBG (HC900 cluster) was downloaded from https://enterobase.readthedocs.io/en/latest/HierCC_lookup.html. A subset of 61 ceBGs with ≥20 pig strains (277,588 strains in total) was used to produce a hierarchical bubble plot (Fig. 1d) showing phylogenetic groupings at subspecies (HC2850), super-lineage (HC2000) and ceBG (HC900) levels, along with pie charts representing source categories. A total of 9,259 genomes from 9 ceBGs, each containing >40% of pig-associated strains, were selected for downstream analysis. In addition, a set of 16,829 genomes from 8 pig-containing ceBGs each containing lower levels (2–40%) of pig-associated strains were also selected and compared with the 9 pig-enriched ceBGs. In addition, 15 Choleraesuis strains were collected by China CDC from northern and eastern regions across China between 2002 and 2022. The DNA of each strain was extracted using the HiPure Bacterial DNA kit (D3146). Paired-end libraries with insert sizes of ~300 bp were prepared following Illumina’s standard genomic DNA library preparation procedure (VAHTS Universal DNA Library Prep kit for Illumina V3) and sequenced on an Illumina NovaSeq 6000 using the S4 reagent kits (v1.5) according to the manufacturer’s instructions.

To show the association between pigs and their enriched *S. enterica* strains, we sequenced the genome of 78 strains of the prominent pig-enriched serovar, Choleraesuis. These include 63 strains from Germany or Austria as part of the University of Warwick/University College Cork 10K genomes project^36^ and 15 from China. They were integrated with public genomes of 679 strains isolated between 1935 and 2022 and one genotype reconstructed from ~1,600-year-old human remains^26^, resulting in a global collection of 757 genomes (Supplementary Table 6) encompassing 41 countries.

### Bioinformatic analysis

The sequencing reads of each strain were quality trimmed using EtoKi prepare^12^, and the high-quality sequences were further assembled into contigs using SPAdes V3.13 (ref. ^37^), which was implemented in the ‘EtoKi assemble’ pipeline. The genes in each assembled genome were predicted and annotated using PROKKA 1.14.6 (ref. ^38^) and had detailed functional predictions using eggnog-mapper v2 (ref. ^39^). The antibiotic resistance genes were predicted using AMRfinder v3.11.14 (ref. ^40^), and the disrupted genes in the pig-enriched populations were predicted using PEPPAN^41^. The multi-sequence alignment for each pig-enriched ceBG was generated using the EtoKi align module and used to build a maximum-likelihood (ML) phylogeny using IQTree v1.6.12 (ref. ^42^) implemented in EtoKi phylo after the removal of recombinant regions using RecHMM^11^.

### Temporal signal and randomization test

The presence of a temporal signal in *Salmonella* serovar Choleraesuis (ceBG1272) was tested using three approaches. The regression of root-to-tip distances and dates of isolation was estimated using TempEst v1.5.3 (ref. ^43^) with a correlation of determination (*R*^2^) of 0.67 and *P* value of 4.15 × 10^−6^. We then randomly permutated the isolation dates of the strains ten times and estimated their *R*^2^ values. The same datasets were also used for BactDating v1.1 (ref. ^44^) inferences as described above, and their substitution rates were compared with the rate from the actual data (Extended Data Fig. 2), showing the presence of a significant temporal signal. We also performed the same tests without the ancient genotype, showing the presence of temporal signals. Furthermore, the tests were also performed for the other eight pig-enriched populations, proving their availability for temporal analyses (Extended Data Fig. 3).

### Population dynamics of serovar Choleraesuis

The ML tree of serovar Choleraesuis was calibrated by dated tips using BEAST^43^ with a GTR substitution model and fixed topology. Eight BEAST runs were prepared by combinations of two clock models of ‘strict clock rate’ and ‘optimized relaxed clock’, and four population models of ‘constant coalescence’, ‘Bayesian skyline’, ‘Birth-death skyline’ and ‘extended Bayesian skyline’. All models were run in ‘Nested Sampling’ mode with eight parallel chains each with ‘chainLength=20000’, ‘particleCount=1’ and ‘subChainLength=5000’. The results were summarized using NSLogAnalyser, and the model with ‘optimized relaxed clock’ and ‘Bayesian skyline’ had the greatest marginal likelihood with a maximum effective sampling size of 632.3. The posterior trees from the best model were then summarized using treeannotator into a maximum clade credibility (MCC) tree and visualized in iTol^45^.

Two downsampling tests were performed by selecting at most ten or five random genomes from each country. We used TreeTime to reconstruct the ancestral states of the internal nodes based on a subtree containing only those selected tips. Each test was run parallel 100 times, and the results were summarized in Extended Data Fig. 4.

### Population dynamics of pig-enriched and pig-containing ceBGs

Furthermore, the population dynamics of each pig-enriched population were estimated using BactDating^46^, which performed Bayesian inference of ancestral dates based on the ML tree. Parallel chains of 5 × 10^6^ samples each were run for each of the substitution models of ‘strictgamma’, ‘mixedgamma’ and ‘carc’. The first 50% of the chain (3 × 10^6^ samples) for each model was discarded as burn-ins, and the convergence of the run was determined by ensuring effective sampling sizes of >100 for all parameters. The results from all samples were compared based on their Bayes factors using the modelcompare function in BactDating, and only the best model for each pig-enriched population was reported. Notably, the dating results for ceBG1272 (Choleraesuis) by BactDating were very similar to that by BEAST, suggesting high reproducibility of the analyses. We then estimated the host transfers and geographic transmissions of each population along the dated trees using the ML algorithm implemented in TreeTime^47^. Similarly, the dates and host transfers of the ancestral nodes in the ML trees of the pig-containing ceBGs were also estimated as described above.

### Transmission events and the correlation between trade data

We define a transmission or host transfer event when the ancestral node and the descending node of a branch are assigned different country and host information. Considering all possible states as *S*, a host transfer or international transmission was counted along the ML tree if the reconstructed states in the ancestral and the descending nodes of a branch were different, and the numbers of host transfers and international transmissions were summarized as ${N}_{\rm{i}\to{j}}$, where *i∈S* and *j∈S* are the states of the ancestral and descending nodes, respectively. Then there is:

$${T}_{\rm{i\to j}}={N}_{\rm{i\to j}}/\sum _{\rm{k\in S}}{N}_{\rm{k\to j}}$$

where ${T}_{\rm{i}\to{j}}$ is the normalized frequency of *Sj* originating from *Si*, *and k∈S* is an iterator for calculating the total number of transmissions into *Sj*. Furthermore, the normalized frequency between two continents *m* and *n* are:

$${\hat{T}}_{{\rm{m}}\to {\rm{n}}}=\sum _{{\rm{a}}\epsilon {\rm{m}}}\sum _{{\rm{b}}\epsilon {\rm{n}}}{T}_{{\rm{a}}\to {\rm{b}}}$$

where *a* and *b* are countries in the continents *m* and *n*, respectively.

The international trade data of all categories were obtained from Harvard Dataverse (https://dataverse.harvard.edu). Trade values were expressed in constant US dollars after adjustment for inflation and summed up. The normalized fluxes of trade between continents were also calculated using a procedure similar to the one mentioned previously. As a result, frequencies of pathogen transmissions and trading among five continents, Asia, Africa, Europe, Oceania and the Americas, were obtained and pairwise Pearson’s correlation coefficients (*R*) between the cross-continental flows of the goods and between the flows of the goods and the pathogens were calculated. All goods and salmonellae were then projected into a two-dimensional space based on their 1 − *R* values using UMAP, which is a non-linear dimension reduction technique that has been extensively used in biological analysis, such as in single-cell studies^48^.

### Reporting summary

Further information on research design is available in the Nature Portfolio Reporting Summary linked to this article.

### Supplementary information

Supplementary Information Supplementary Tables 1–4.

Reporting Summary

Supplementary Tables Supplementary Tables 5 and 6.

### Source data

Source Data Fig. 1 Statistical source data.

Source Data Fig. 2 Statistical source data.

Source Data Fig. 3 and Extended Data Figs. 5 and 6 Statistical source data.

Source Data Fig. 4 and Extended Data Figs. 7 and 8 Statistical source data.

Source Data Extended Data Figs. 1–4 Statistical source data.

## Data Availability

The raw sequencing reads for the 15 Chinese strains have been deposited in the Genome Sequence Archive in the National Genomics Data Center, China National Center for Bioinformation/Beijing Institute of Genomics, Chinese Academy of Sciences (GSA: CRA012579) and are publicly accessible at https://ngdc.cncb.ac.cn/gsa. The assembled genome sequences have been deposited in the Genome Warehouse (GWH) in the National Genomics Data Center with BioProject accession PRJCA019682. The raw reads for 67 European Choleraesuis strains were deposited in Short Reads Archive (SRA) at EBI under BioProject accession PRJEB20997, as part of the University of Warwick/University College Cork (UOWUCC) 10K genomes project. A detailed list of the sample accession codes for all Choleraesuis strains is available in Supplementary Table 6. Assembled genomes for all pig-enriched populations were available as a workspace in EnteroBase at https://enterobase.warwick.ac.uk/a/100355. The resulting figures and underlying data of 61 ceBGs with ≥20 pig strains are all available at https://observablehq.com/d/232a986be1a99113. Source data are provided with this paper.
