# Supplementary material for: Centralized industrialization of pork in Europe and America contributes to the global spread of Salmonella enterica
Source: Nat Food. 2024 May 9;5(5):413–22. doi: 10.1038/s43016-024-00968-1 (PMC11132987; doi:10.1038/s43016-024-00968-1)
Supplement: Supplementary file 1 — Supplementary Tables 1–4. [file 43016_2024_968_MOESM1_ESM.pdf]

nature food

Article https://doi.org/10.1038/s43016-024-00968-1

Centralized industrialization of pork in Europe and America contributes to the global spread of *Salmonella enterica*

In the format provided by the authors and unedited

Supplementary Information

Supplementary Tables 2
Table S1 Pig-enriched HC5s that were isolated from multiple countries 2
Table S2 Simpson's diversity of clades at the national level 6
Table S3 Distribution of the colistin-resistant genes in Choleraesuis 7
Table S4 The frequencies of transmissions in each pair of regions across all 100 random downsamplings 8

Supplementary Tables

Table S1 Pig-enriched HC5s that were isolated from multiple countries

HC5s	HC900s	Category	Continents (Country:No.)	Country	Sources
67540	HC900_3 (Derby)	Cross-continent	Europe (UK:2); North America (US:1,Canada:1); South America(Ecuador:2)	United Kingdom; United States; Canada; Ecuador	Pig:2, Human:4
87053	HC900_3 (Derby)	Cross-continent	Asia (China:3); North America (US:1)	China; United States	Pig:2, Human:2
88501	HC900_3 (Derby)	Cross-continent	North America(DO:2, US:1); Europe (Italy:1)	Dominican Republic; United States; Italy	Pig:2, Human:2
114252	HC900_3 (Derby)	Cross-continent	Africa (Nigeria: 3); Europe (Ireland:1)	Nigeria; Ireland	Pig:3, Human:1
2895	HC900_10 (Adelaide)	Cross-continent	Europe (UK:1); North America (US:25)	United Kingdom; United States	Pig:6, Human:16, Bovine:1, Food/Env:3
41634	HC900_10 (Adelaide)	Cross-continent	Europe (UK:1); North America (US:54)	United Kingdom; United States	Pig:31, Human:17, Bovine:4, Food/Env:3
228118	HC900_17 (Chailey)	Cross-continent	North America (US:15); Oceania (Australia:1)	United States; Australia	Pig:14, Human:1
16822	HC900_35 (Worthington)	Cross-continent	North America (US:2); South America (Brazil:2)	United States; Brazil	Pig:2, Bovine:2
4941	HC900_37 (London)	Cross-continent	Asia (China:2); North America (US:42)	China; United States	Pig:28, Human:8, Bovine:1, Poultry:3, Food/Env:2
119330	HC900_37 (London)	Cross-continent	Asia (China:4); North America (US:1)	China; United States	Pig:1, Human:3, Bovine:1

157423	HC900_37 (London)	Cross-continent	North America (US:3); South America (Brazil:1)	United States; Brazil	Pig:3, Bovine:1
254629	HC900_37 (London)	Cross-continent	Asia (Vietnam:1); Oceania (Australia:1)	Vietnam; Australia	Pig:1, Human:1
269496	HC900_37 (London)	Cross-continent	Asia (Vietnam:1); North America (Canada:1)	Vietnam; Canada	Pig:1, Human:1
178648	HC900_621 (Ohio)	Cross-continent	Africa (Nigeria:1); North America (US:1)	Nigeria; United States	Pig:1, Human:1
30373	HC900_1272 (Choleraesuis)	Cross-continent	Europe (Germany:1); North America (US:2)	Germany; United States	Pig:2, Food/Env:1
531	HC900_3 (Derby)	Intra-continent	North America (US:12,Canada:2)	United States; Canada	Pig:7, Human:2, Food/Env:4
8503	HC900_3 (Derby)	Intra-continent	North America (US:1,Canada:1)	United States; Canada	Pig:2
54053	HC900_3 (Derby)	Intra-continent	Europe (UK:1, Denmark:1)	United Kingdom; Denmark	Pig:1, Food/Env:1
62891	HC900_3 (Derby)	Intra-continent	North America (US:1, Canada:1)	United States; Canada	Pig:2
78993	HC900_3 (Derby)	Intra-continent	Europe(Ireland:8, UK:1)	Ireland; Northern Ireland	Pig:7, Bovine:1
91621	HC900_3 (Derby)	Intra-continent	North America (US:3,Canada:1)	United States; Canada	Pig:1, Human:3
102129	HC900_3 (Derby)	Intra-continent	Europe(Denmark:1, Poland:2)	Denmark; Poland	Pig:2

102206	HC900_3 (Derby)	Intra-continent	Europe(Denmark:1, Ireland:1)	Denmark; Ireland	Pig:1, Food/Env:1
102841	HC900_3 (Derby)	Intra-continent	Europe(Germany:1, Netherlands:1, Poland:2)	Germany; Netherlands; Poland	Pig:2, Human:1, Food/Env:1
146979	HC900_3 (Derby)	Intra-continent	North America (US:1,Canada:1)	United States; Canada	Pig:2
151156	HC900_3 (Derby)	Intra-continent	Asia(Cambodia:1, Thailand:1)	Cambodia; Thailand	Pig:2
175100	HC900_3 (Derby)	Intra-continent	Europe(Ireland:3, UK:1)	Ireland; Northern Ireland	Pig:4
64445	HC900_10 (Adelaide)	Intra-continent	North America (US:11, Mexico:1)	United States; Mexico	Pig:9, Human:1, Food/Env:2
68622	HC900_35 (Worthington)	Intra-continent	North America (US:1,Canada:2)	United States; Canada	Pig:1, Bovine:1, Food/Env:1
156927	HC900_621 (Ohio)	Intra-continent	North America (US:1,Canada:1)	United States; Canada	Pig:2
4432	HC900_1272 (Choleraesuis)	Intra-continent	Europe (Poland:4,Germany:1)	Poland; Germany	Pig:4, Human:1
44098	HC900_1272 (Choleraesuis)	Intra-continent	Europe (Denmark:1, France:1)	Denmark; France	Pig:2
164515	HC900_1272 (Choleraesuis)	Intra-continent	North America (US:22, Mexico:1)	United States; Mexico	Pig:23
168350	HC900_1272 (Choleraesuis)	Intra-continent	Europe (Czech Republic:3, Germany:25, Italy:1)	Czech Republic; Germany; Italy	Pig:12, Human:16, Food/Env:1

222280	HC900_1272 (Choleraesuis)	Intra-continent	Europe (Austria:1, Slovenia:11)	Austria; Slovenia	Pig:11, Human:1

Note: The item in the table including HC5 cluster from Enterobase, corresponded with pig-enriched ceBGs (HC900), continent, transnational, and host sources information.

Table S2 Simpson's diversity of clades at the national level

Country	Simpson's diversity index
France	0.781818182
United Kingdom	0.750445633
Thailand	0.678571429
Poland	0.659340659
Germany	0.639900799
China	0.60960961
Austria	0.54500684
Italy	0.452173913
Canada	0.416666667
Denmark	0.410752688
Brazil	0.285714286
United States	0.153553632
Vietnam	0.117647059
Estonia	0
Lithuania	0
Russia	0
Slovenia	0

Table S3 Distribution of the colistin-resistant genes in Choleraesuis

ID	Collection Year	Source Simple	Country	Continent	mcr-1.1	mcr-3.1	mcr-3.40
9709	2014	Human	China	East Asia	0	1	0
SAL_FB4951AA	2017	Pig	Brazil	South America	1	0	0
SAL_GA0174AA		Human			0	1	0
SAL_IB1606AA	2017	Human	Germany	West Europe	0	0	1
SAL_OA2121AA	2015	Human	United Kingdom	West Europe	0	1	0
SAL_OA2122AA	2015	Human	United Kingdom	West Europe	0	1	0
SAL_OA2126AA	2016	Human	United Kingdom	West Europe	0	1	0
SAL_PA8568AA	2016	Human	United Kingdom	West Europe	0	1	0

Table S4 The frequencies of transmissions in each pair of countries/regions across all 100 random downsamplings

5 in each country/region		
**Source**	**Target**	**Frequency**
Europe	Argentina	200
Estonia	Europe	164
Europe	France	163
NEurope	United_States	160
NEurope	Asia	125
NEurope	Americas	120
Americas	France	106
Americas	United_States	102
Vietnam	Asia	101
Vietnam	Canada	100
Hungary	Italy	100
Africa	Russia	100
Cameroon	Argentina	100
Africa	Cameroon	100
Europe	Canada	100
United_States	Americas	99
Vietnam	Chinese_Mainland	96
NEurope	Canada	95
Africa	Denmark	93
NEurope	Africa	93
Czech_Republic	Germany	91

10 in each country/region		
**Source**	**Target**	**Frequency**
Europe	France	231
United_States	Americas	214
Europe	Argentina	200
NEurope	United_States	167
United_States	NEurope	154
Estonia	Europe	151
Czech_Republic	Germany	146
Africa	United_Kingdom	128
United_Kingdom	Peru	128
Chinese_Mainland	Austria	126
Austria	Slovenia	106
Africa	Chinese_Mainland	105
Cameroon	Argentina	100
Europe	Brazil	100
Europe	Canada	100
Vietnam	Asia	100
Vietnam	Brazil	100
Vietnam	Canada	100
Africa	Cameroon	99
Africa	Russia	99
Chinese_Mainland	NEurope	99

Africa	Europe	91
Europe	Brazil	90
Chinese_Mainland	NEurope	90
Denmark	Germany	84
Americas	NEurope	82
NEurope	Chinese_Taiwan	80
United_Kingdom	Australia	78
Africa	Lithuania	78
Chinese_Mainland	Austria	78
Cameroon	Poland	77
Africa	United_Kingdom	77
Americas	Australia	74
Austria	Slovenia	68
Vietnam	Brazil	64
NEurope	Italy	63
Slovenia	Austria	61
Vietnam	Peru	61
Peru	Vietnam	60
Slovenia	Hungary	57
NEurope	Peru	56
Hungary	Slovenia	55
Poland	Czech_Republic	54
Estonia	Hungary	54
Austria	Hungary	53
NEurope	United_Kingdom	49

Denmark	Germany	98
Hungary	Italy	98
Vietnam	Chinese_Mainland	96
Americas	France	95
Africa	Europe	94
United_States	France	93
Chinese_Mainland	United_Kingdom	92
United_Kingdom	Australia	92
Chinese_Mainland	Asia	91
NEurope	Asia	91
United_Kingdom	Chinese_Mainland	91
Africa	Denmark	90
Poland	Czech_Republic	88
Denmark	Poland	84
Slovenia	Austria	82
Africa	Asia	79
Hungary	Slovenia	78
Slovenia	Hungary	78
Vietnam	France	76
United_States	Chinese_Taiwan	74
Italy	United_Kingdom	70
NEurope	Italy	69
France	Denmark	68
NEurope	Canada	68
Cameroon	Poland	67

United_Kingdom	Peru	49
Americas	Denmark	48
Africa	Chinese_Mainland	47
NEurope	Estonia	47
Czech_Republic	Poland	46
Vietnam	France	45
Africa	Asia	44
Chinese_Mainland	United_Kingdom	42
Americas	Poland	41
Estonia	Germany	39
Lithuania	Poland	36
Chinese_Mainland	Asia	36
Hungary	Poland	35
Estonia	Poland	35
Italy	United_Kingdom	35
Denmark	Poland	33
Peru	United_Kingdom	33
Denmark	France	32
NEurope	France	31
Estonia	Slovenia	30
Estonia	Austria	30
United_Kingdom	Chinese_Mainland	29
Asia	Chinese_Mainland	28
Australia	United_Kingdom	27
Chinese_Mainland	Italy	27

France	Americas	67
Hungary	Poland	67
Lithuania	Poland	67
NEurope	Africa	66
France	Poland	62
United_Kingdom	Vietnam	61
Denmark	France	59
Americas	United_States	58
United_States	United_Kingdom	58
Austria	Hungary	57
Chinese_Mainland	Italy	57
NEurope	Americas	56
United_Kingdom	NEurope	56
Italy	Germany	54
NEurope	France	54
Chinese_Mainland	Lithuania	53
United_States	Australia	52
Australia	United_Kingdom	50
Estonia	Poland	49
Lithuania	United_Kingdom	47
Poland	Austria	47
Africa	Lithuania	46
Estonia	Hungary	46
Poland	France	45
France	United_States	44

United_States	Australia	25
Austria	Czech_Republic	25
Lithuania	United_Kingdom	25
Poland	Austria	23
Poland	Slovenia	23
United_States	NEurope	23
United_States	France	22
Italy	Germany	22
Hungary	Austria	22
Hungary	Germany	21
United_Kingdom	Vietnam	21
Chinese_Mainland	Lithuania	21
United_States	Chinese_Taiwan	20
France	Poland	19
Vietnam	United_Kingdom	19
Chinese_Mainland	Germany	18
Russia	Austria	18
Slovenia	Czech_Republic	17
Cameroon	Denmark	16
United_States	United_Kingdom	16
NEurope	Hungary	16
France	Denmark	16
Poland	Germany	15
Vietnam	Austria	15
United_Kingdom	NEurope	15

Estonia	Germany	42
NEurope	Estonia	40
Poland	Slovenia	40
Americas	NEurope	39
Chinese_Mainland	Peru	39
United_States	Italy	38
Chinese_Mainland	Germany	35
Czech_Republic	Poland	35
France	NEurope	35
Hungary	Germany	35
Poland	Germany	35
Russia	Austria	35
NEurope	United_Kingdom	34
Cameroon	Denmark	33
Italy	Austria	31
Americas	Australia	30
United_Kingdom	United_States	30
United_States	Canada	30
Poland	Denmark	29
Europe	United_Kingdom	27
NEurope	Chinese_Taiwan	26
Slovenia	Estonia	26
Slovenia	Germany	25
Americas	Poland	23
Slovenia	Poland	23

Slovenia	Germany	14
Europe	United_Kingdom	14
Austria	Poland	13
Austria	Germany	13
Africa	France	12
Italy	Hungary	12
NEurope	Austria	12
Slovenia	Estonia	12
Italy	Europe	12
Chinese_Mainland	Australia	12
Italy	Estonia	12
NEurope	Slovenia	12
Hungary	Estonia	12
United_Kingdom	Asia	11
Italy	Austria	11
NEurope	Germany	11
Americas	United_Kingdom	11
Czech_Republic	Italy	11
Slovenia	Europe	11
Chinese_Mainland	Peru	11
Italy	France	10
Chinese_Mainland	United_States	10
Americas	Germany	10
United_States	Italy	10
United_States	Peru	10

United_States	Africa	23
Czech_Republic	Italy	22
France	Germany	22
Italy	Europe	22
Italy	France	22
United_States	Poland	22
Chinese_Mainland	Vietnam	21
Estonia	Austria	21
Estonia	Slovenia	21
Austria	Germany	20
Chinese_Mainland	United_States	20
NEurope	Slovenia	20
Slovenia	Europe	20
United_Kingdom	Asia	20
United_Kingdom	Germany	20
Slovenia	United_Kingdom	19
United_States	Asia	19
Hungary	Austria	18
Vietnam	Peru	18
Americas	Denmark	17
France	Australia	17
France	United_Kingdom	16
Germany	Austria	16
Italy	Estonia	16
Italy	Hungary	16

France	Brazil	10
Poland	France	9
Austria	Chinese_Mainland	9
Slovenia	Poland	9
United_Kingdom	Europe	9
Poland	Denmark	8
NEurope	Vietnam	8
Austria	Estonia	8
Germany	Denmark	8
United_Kingdom	United_States	8
Austria	Europe	7
France	Americas	7
Australia	United_States	7
France	United_States	7
Estonia	United_Kingdom	7
Austria	NEurope	7
Austria	United_Kingdom	7
United_States	Poland	7
United_Kingdom	Germany	7
Peru	Chinese_Mainland	7
Austria	Italy	7
Africa	Germany	7
Germany	Poland	7
United_States	Africa	6
Asia	NEurope	6

United_States	Denmark	16
Italy	Poland	15
United_States	Peru	14
Africa	NEurope	13
United_States	Austria	13
Vietnam	United_Kingdom	13
Poland	United_Kingdom	12
United_States	Vietnam	12
Asia	United_States	11
Czech_Republic	Austria	11
Germany	United_Kingdom	11
Lithuania	Austria	11
Africa	Germany	10
Austria	Poland	10
Germany	Denmark	10
Austria	United_Kingdom	9
Denmark	United_Kingdom	9
Estonia	United_Kingdom	9
Germany	France	9
United_States	Chinese_Mainland	9
Vietnam	United_States	9
Americas	Austria	8
Austria	Czech_Republic	8
Austria	Estonia	8
United_Kingdom	Africa	8

United_States	Denmark	6
Vietnam	United_States	6
Vietnam	Germany	6
Poland	Hungary	6
United_States	Asia	6
Czech_Republic	Austria	6
Americas	Austria	6
Italy	Poland	6
United_States	Germany	5
United_States	Canada	5
United_States	Vietnam	5
Lithuania	Austria	5
Germany	United_Kingdom	5
Germany	Italy	5
NEurope	Chinese_Mainland	5
Chinese_Mainland	Vietnam	5
Italy	Slovenia	5
United_States	Austria	5
Lithuania	Chinese_Mainland	5
United_States	Estonia	4
Australia	Austria	4
Vietnam	Australia	4
Germany	Estonia	4
Peru	Australia	4
Peru	United_States	4

Vietnam	Austria	8
Australia	United_States	7
Chinese_Mainland	Australia	7
Germany	Poland	7
NEurope	Austria	7
United_States	Germany	7
Asia	Chinese_Mainland	6
Austria	Italy	6
Peru	Vietnam	6
Slovenia	Italy	6
United_Kingdom	Europe	6
United_States	Estonia	6
United_States	Slovenia	6
Asia	United_Kingdom	5
Austria	Chinese_Mainland	5
Austria	Europe	5
Austria	NEurope	5
Italy	Slovenia	5
Slovenia	France	5
Americas	Germany	4
NEurope	Germany	4
Slovenia	Czech_Republic	4
Americas	United_Kingdom	3
Australia	Austria	3
Austria	France	3

Denmark	United_Kingdom	3	France	Africa	3
Africa	Poland	3	France	Austria	3
Germany	Austria	3	Germany	Estonia	3
France	Australia	3	Germany	Hungary	3
France	Germany	3	Germany	Slovenia	3
Hungary	Czech_Republic	3	NEurope	Chinese_Mainland	3
Africa	United_States	3	Africa	Poland	2
Germany	Hungary	3	Australia	Chinese_Mainland	2
Germany	Europe	3	Denmark	United_States	2
Asia	United_States	3	Europe	Germany	2
Australia	Germany	3	France	Canada	2
Hungary	Europe	2	France	Chinese_Mainland	2
Slovenia	United_Kingdom	2	Germany	Europe	2
France	NEurope	2	Germany	NEurope	2
France	United_Kingdom	2	Poland	United_States	2
Asia	Vietnam	2	United_Kingdom	Slovenia	2
United_States	Chinese_Mainland	2	Africa	France	1
Denmark	United_States	2	Africa	United_States	1
United_Kingdom	Italy	2	Austria	Americas	1
Europe	Estonia	1	Austria	Australia	1
Poland	United_States	1	Estonia	Italy	1
Poland	United_Kingdom	1	France	Peru	1
Austria	United_States	1	Germany	Chinese_Mainland	1
Australia	Chinese_Mainland	1	Germany	Czech_Republic	1
Germany	France	1	Germany	Italy	1

Estonia	Italy	1
Europe	Germany	1
Asia	United_Kingdom	1
Slovenia	France	1
Germany	Asia	1
Africa	NEurope	1
Germany	Chinese_Mainland	1
Europe	Poland	1
Estonia	Czech_Republic	1
Poland	Europe	1
Germany	NEurope	1
Chinese_Mainland	Africa	1
United_Kingdom	Lithuania	1
Germany	Slovenia	1
Germany	United_States	1
Hungary	Estonia	1
NEurope	Denmark	1
NEurope	Hungary	1
Peru	United_Kingdom	1
Poland	Hungary	1
United_Kingdom	Cameroon	1
United_Kingdom	Italy	1
United_Kingdom	Lithuania	1
United_Kingdom	Russia	1
Vietnam	Australia	1
Vietnam	Germany	1
