# Supplementary material for: Centralized industrialization of pork in Europe and America contributes to the global spread of Salmonella enterica
Source: Nat Food. 2024 May 9;5(5):413–22. doi: 10.1038/s43016-024-00968-1 (PMC11132987; doi:10.1038/s43016-024-00968-1)
Supplement: Supplementary file 2 — Reporting Summary [file 43016_2024_968_MOESM2_ESM.pdf]

nature portfolio

Corresponding author(s): Zhemin Zhou, Biao Kan, Yi Ren, Xin Lu

Last updated by author(s): Jan 17, 2024

Reporting Summary

Nature Portfolio wishes to improve the reproducibility of the work that we publish. This form provides structure for consistency and transparency in reporting. For further information on Nature Portfolio policies, see our Editorial Policies and the Editorial Policy Checklist.

Statistics

For all statistical analyses, confirm that the following items are present in the figure legend, table legend, main text, or Methods section.

n/a	Confirmed	
☐	☒	The exact sample size (*n*) for each experimental group/condition, given as a discrete number and unit of measurement
☒	☐	A statement on whether measurements were taken from distinct samples or whether the same sample was measured repeatedly
☐	☒	The statistical test(s) used AND whether they are one- or two-sided<br>*Only common tests should be described solely by name; describe more complex techniques in the Methods section.*
☒	☐	A description of all covariates tested
☐	☒	A description of any assumptions or corrections, such as tests of normality and adjustment for multiple comparisons
☐	☒	A full description of the statistical parameters including central tendency (e.g. means) or other basic estimates (e.g. regression coefficient) AND variation (e.g. standard deviation) or associated estimates of uncertainty (e.g. confidence intervals)
☐	☒	For null hypothesis testing, the test statistic (e.g. *F*, *t*, *r*) with confidence intervals, effect sizes, degrees of freedom and *P* value noted<br>*Give P values as exact values whenever suitable.*
☐	☒	For Bayesian analysis, information on the choice of priors and Markov chain Monte Carlo settings
☐	☒	For hierarchical and complex designs, identification of the appropriate level for tests and full reporting of outcomes
☐	☒	Estimates of effect sizes (e.g. Cohen's *d*, Pearson's *r*), indicating how they were calculated

Our web collection on statistics for biologists contains articles on many of the points above.

Software and code

Policy information about availability of computer code

Data collection	No software was used for data collection.
Data analysis	The following software were used in the analysis: Enterobase v1.2.0, EToKi v1.3, SPAdes v3.13, PROKKA v1.14.6, TempEst v1.5.3, BactDating v1.1, AMRfinder v3.11.14, eggnog-mapper v2, PEPPAN v1.0.5, IQTree v1.6.12, TreeTime v0.9.6, BEAST v2, iTOL v6, RecHMM v1, UMAP v3, R package "skygrowth", Python 3.10.12.

For manuscripts utilizing custom algorithms or software that are central to the research but not yet described in published literature, software must be made available to editors and reviewers. We strongly encourage code deposition in a community repository (e.g. GitHub). See the Nature Portfolio guidelines for submitting code & software for further information.

Data

Policy information about availability of data

All manuscripts must include a data availability statement. This statement should provide the following information, where applicable:

- Accession codes, unique identifiers, or web links for publicly available datasets
- A description of any restrictions on data availability
- For clinical datasets or third party data, please ensure that the statement adheres to our policy

Our analysis used global trade data from the Harvard database(https://doi.org/10.7910/DVN/HOOTBB),The raw sequencing reads for the 15 Chinese strains have been deposited in the Genome Sequence Archive in the National Genomics Data Center, China National Center for Bioinformation / Beijing Institute of Genomics,

Chinese Academy of Sciences (GSA: CRA012579) and are publicly accessible at https://ngdc.cncb.ac.cn/gsa. The assembled genome sequences have been deposited in the Genome Warehouse (GWH) in the National Genomics Data Center with bioproject accession PRJCA019682. The raw reads for 67 European Choleraesuis strains were deposited in Short Reads Archive (SRA) at EBI under BioProject accession: PRJEB20997, as part of the University of Warwick/University College Cork (UOWUCC) 10K genomes project. A detailed list of the sample accession codes for all Choleraesuis strains is available in Additional Supplementary Files Table 2. Assembled genomes for all pig-enriched populations were available as a workspace in EnteroBase at https://enterobase.warwick.ac.uk/a/100355. The resulting figures and underlying data are all available at http:// observablehq.com/d/232a986be1a99113.

Human research participants

Policy information about studies involving human research participants and Sex and Gender in Research.

Reporting on sex and gender	*Use the terms sex (biological attribute) and gender (shaped by social and cultural circumstances) carefully in order to avoid confusing both terms. Indicate if findings apply to only one sex or gender; describe whether sex and gender were considered in study design whether sex and/or gender was determined based on self-reporting or assigned and methods used. Provide in the source data disaggregated sex and gender data where this information has been collected, and consent has been obtained for sharing of individual-level data; provide overall numbers in this Reporting Summary. Please state if this information has not been collected. Report sex- and gender-based analyses where performed, justify reasons for lack of sex- and gender-based analysis.*
Population characteristics	*Describe the covariate-relevant population characteristics of the human research participants (e.g. age, genotypic information, past and current diagnosis and treatment categories). If you filled out the behavioural & social sciences study design questions and have nothing to add here, write "See above."*
Recruitment	*Describe how participants were recruited. Outline any potential self-selection bias or other biases that may be present and how these are likely to impact results.*
Ethics oversight	*Identify the organization(s) that approved the study protocol.*

Note that full information on the approval of the study protocol must also be provided in the manuscript.

Field-specific reporting

Please select the one below that is the best fit for your research. If you are not sure, read the appropriate sections before making your selection.

☒ Life sciences ☐ Behavioural & social sciences ☐ Ecological, evolutionary & environmental sciences

For a reference copy of the document with all sections, see nature.com/documents/nr-reporting-summary-flat.pdf

Life sciences study design

All studies must disclose on these points even when the disclosure is negative.

Sample size	For systematic investigation of all Salmonella enterica populations, we collected a total of 362,931 S. enterica strains from Enterobase up to July 2022. For the population dynamics analysis of pig-associated Salmonella, we collected 9259 pig-enriched ceBGs genomes and 16829 pig-containing ceBGs genomes for comparison. In addition, 15 Choleraesuis strains were collected by the China CDC from northern and eastern regions across China between 2002 and 2022. The sample size for this study was determined by collecting all available genomes of Salmonella up to the present, aiming to achieve a comprehensive understanding of the bacterium. While it may not be exhaustive, this represents the best achievable result with current resources.
Data exclusions	We removed genomes with incomplete temporal and geographic information in the phylogeographic analysis of all pig-enriched Salmonella serovars. We further removed the ancient genotype of serovar Choleraesuis in temporal analysis for Extended Data Figure 3.
Replication	We conducted two random downsampling tests with a maximum of five or ten strains from each country and estimated the ancestral states along the dated tree using TreeTime. Each test was run in 100 parallel. The results showed that the ancestral nodes were still predominantly assigned to North European countries. The only exception is Clade 2.1, which is estimated to be from Africa. We attributed this to a cluster of Cameroon isolates near the root of the Clade 2.1. The downsampling process removed the majority of the strains from the UK and Germany because they were genetically more divergent (by the Simpson indices) but kept the Cameroon isolates because they all fell into the only cluster there.
Randomization	To examine the impact of sampling bias on the reconstruction of ancestral geographic states, we employed the random.sample() function in Python to randomly select up to 5 or 10 strains from each country or region. Additionally, to confirm the presence of temporal signal, we utilized the random.shuffle() function in Python to randomly permute the isolation dates of the strains. The results obtained from these script executions were directly used for analysis, devoid of any human intervention.
Blinding	The investigators were blinded to group allocation during data analysis as the grouping process was entirely automated and conducted by a Python script. Therefore, blinding was maintained throughout the group allocation, ensuring objectivity and minimizing potential biases.

Reporting for specific materials, systems and methods

We require information from authors about some types of materials, experimental systems and methods used in many studies. Here, indicate whether each material, system or method listed is relevant to your study. If you are not sure if a list item applies to your research, read the appropriate section before selecting a response.

Materials & experimental systems

n/a	Involved in the study
☒	☐ Antibodies
☒	☐ Eukaryotic cell lines
☒	☐ Palaeontology and archaeology
☒	☐ Animals and other organisms
☒	☐ Clinical data
☒	☐ Dual use research of concern

Methods

n/a	Involved in the study
☒	☐ ChIP-seq
☒	☐ Flow cytometry
☒	☐ MRI-based neuroimaging
